# Molecular Design in Practice: A Review of Selected Projects in a French Research Institute That Illustrates the Link between Chemical Biology and Medicinal Chemistry

**DOI:** 10.3390/molecules26196083

**Published:** 2021-10-08

**Authors:** Benoit Deprez, Damien Bosc, Julie Charton, Cyril Couturier, Rebecca Deprez-Poulain, Marion Flipo, Florence Leroux, Baptiste Villemagne, Nicolas Willand

**Affiliations:** 1 Univ. Lille, Inserm, Institut Pasteur Lille, U1177-Drugs and Molecules for Living Systems, F-59000 Lille, France; damien.bosc@univ-lille.fr (D.B.); julie.charton@univ-lille.fr (J.C.); cyril.couturier@univ-lille.fr (C.C.); rebecca.deprez@univ-lille.fr (R.D.-P.); marion.flipo@univ-lille.fr (M.F.); florence.leroux@pasteur-lille.fr (F.L.); baptiste.villemagne@univ-lille.fr (B.V.); 2 Univ. Lille, CNRS, Inserm, CHU Lille, Institut Pasteur de Lille, US 41-UMS 2014-PLBS, F-59000 Lille, France

**Keywords:** drug discovery, hit to lead optimization, target engagement, target validation, kinetic target guided synthesis, fragment, early ADME

## Abstract

Chemical biology and drug discovery are two scientific activities that pursue different goals but complement each other. The former is an interventional science that aims at understanding living systems through the modulation of its molecular components with compounds designed for this purpose. The latter is the art of designing drug candidates, i.e., molecules that act on selected molecular components of human beings and display, as a candidate treatment, the best reachable risk benefit ratio. In chemical biology, the compound is the means to understand biology, whereas in drug discovery, the compound is the goal. The toolbox they share includes biological and chemical analytic technologies, cell and whole-body imaging, and exploring the chemical space through state-of-the-art design and synthesis tools. In this article, we examine several tools shared by drug discovery and chemical biology through selected examples taken from research projects conducted in our institute in the last decade. These examples illustrate the design of chemical probes and tools to identify and validate new targets, to quantify target engagement in vitro and in vivo, to discover hits and to optimize pharmacokinetic properties with the control of compound concentration both spatially and temporally in the various biophases of a biological system.

## 1. Introduction

Understanding biological processes implies a comprehensive and dynamic view of living systems at molecular and atomic scales. This knowledge can be acquired through a combination of observational and experimental (i.e., interventional) science. Eventually, it is used to design therapeutic interventions using molecules specifically designed to promote a health state of the organism. As both the biological components and the tools to intervene are chemical by nature, chemical sciences are key to making biological experiments not only informative but also interpretable, and knowledge relevant to a therapeutic action. Chemical biology and drug discovery are two scientific activities based on chemical sciences applied to chemical and biological objects. The former aims at describing biological processes through chemically controlled molecular intervention and the latter uses the knowledge on biological processes to design molecules that drive the cell and organism states in a chemically controlled way. In both activities, specific molecular interactions between biological components and xenobiotics are sought. In this paper, we illustrate the use of these concepts in drug discovery through real life examples taken at the different steps of target and drug discovery: target selection, validation, and engagement; hit discovery; hit-to-lead; and lead optimization, especially by fine-tuning ADME properties.

## 2. Target Validation and Engagement

### 2.1. Chemical Biology Approaches to Reprogram the Transcriptome of Bacteria and Select Drug Candidates

Target validation and target engagement are the two pillars of drug development to ensure in vivo action and a potential therapeutic benefit.

**Figure 1:**
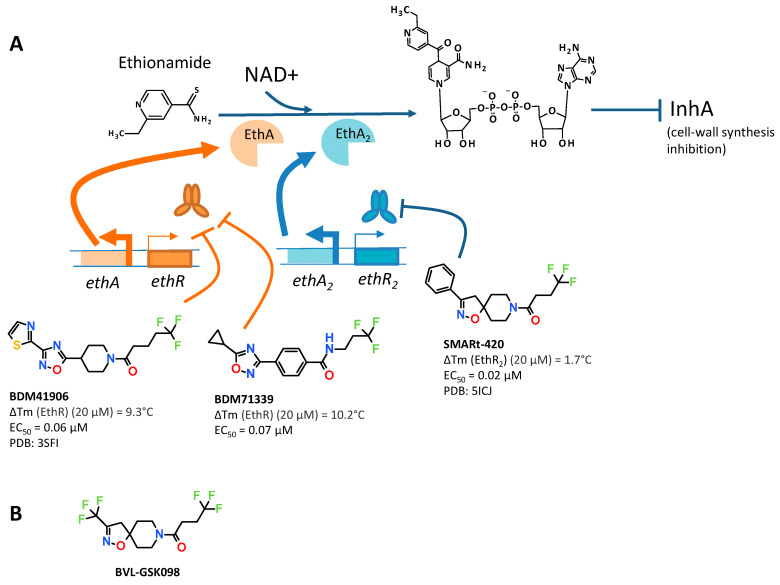
(**A**) Ethionamide bioactivation pathways: EthA and EthA2 are triggered by the inhibition of mycobacterial transcription factors EthR and EthR_2_ with small molecules BDM41906, BDM71339, and SMARt-420; (**B**) structure of the clinical candidate BVL-GSK098.

Tuberculosis (TB) caused by *Mycobacterium tuberculosis* still remains a huge global health challenge, particularly considering the fact that about one-third of the world’s population has latent TB, as currently estimated by the World Health Organization. One of the unique features shared by many TB drugs currently used in the first- and second-line treatments is that they behave like a trojan horse. Indeed, their antibacterial activity is only brought to light once they are transformed inside the bacteria by its own enzymatic machinery [1]. Most of the bioactivation processes involved have been studied and understood since the 2000s. For example, the two drugs ethionamide (ETH) and isoniazid (INH), which are, respectively, the substrates of two different oxidative enzymes: EthA, a Baeyer–Villiger mono-oxygenase; and KatG, a catalase peroxidase; are transformed into two distinct NAD^+^ adducts. These adducts then inhibit the same target called InhA, a NADH-dependent enoyl-acyl-carrier-protein reductase involved in the biosynthesis of mycolic acids which are major components of the mycobacterial cell-wall (illustrated in Figure 1A for ETH). Not surprisingly, the most frequent resistance mechanism occurs by mutation in enzymes involved in these bioactivation pathways [2].

In physiological conditions, the in vitro and in vivo antimycobacterial potency of thioamide drugs as ethionamide and prothionamide is limited; indeed, their bioactivation pathway is negatively regulated by a transcription factor called EthR. More importantly, the silencing of this gene was shown to improve the susceptibility of *M. bovis BCG* to ethionamide [3]. It therefore appeared fundamental from a therapeutic point of view to understand whether it was possible to reprogram the transcriptome of the bacteria by targeting EthR with small organic molecules or not. Two parallel strategies, a screening of lead-like molecules based on the X-ray structure of the repressor on one hand and a fragment-based optimization on the other hand, led us to the discovery of two families of potent inhibitors of EthR with a common central 1,2,4-oxadiazole core [4,5,6,7]. The binding mode of this family of boosters to EthR was then intensively studied by co-crystallization and X-ray diffraction, and the engagement of the target in bacterio proved to greatly improve the expression of EthA and thus the bioactivation of ethionamide. Through the synthesis of more than 500 molecules, two compounds were selected for further in vivo studies: BDM41906 and BDM71339 (Figure 1A). The co-administration of ETH with BDM41906, given orally at 20 mg/kg, reduced the mycobacterial load as effectively as a three-times-higher dose of ETH monotherapy [8]. BDM71339 also proved to be successfully active in vivo [9]. At this stage, we had just shown that targeting mycobacterial transcriptional repressor such as EthR with small molecules did indeed make the bacteria more sensitive to ethionamide.

The major shift within the framework of this project occurred when we performed the key replacement of the oxadiazole-piperidine moiety by a spiroisoxazoline motif. In fact, it turned out that this modification caused a loss of the binding to EthR without causing a loss of ethionamide activity boosting on the bacteria. The transcriptomic analysis with our lead compound SMARt-420 allowed us to discover that this booster triggers a different transcription factor called EthR_2_, thus awakening new alternative ETH activation pathways leading to the reversion of clinical resistant strains (Figure 1A) [10].

SMARt-420 therefore represents a good example of a chemical probe that allows the reprogramming of the transcriptome of *M. tuberculosis*. Moreover, the optimization of this chemical family led to the discovery of the clinical candidate BVL-GSK098 (Figure 1B). BVL-GSK098 entered Phase 1 development with first-subject-first-visit on November 27, 2020. This work has paved the way for the development of bacterial transcriptional regulators in drug development for bacterial infections to circumvent antimicrobial resistance; BVL-GSK098 is the first example to reach the clinic.

### 2.2. Chemical Biology Strategies to Quantify Target Engagement

Along with target validation, target engagement in living cells is crucial for the development of new drugs.

**Figure 2:**
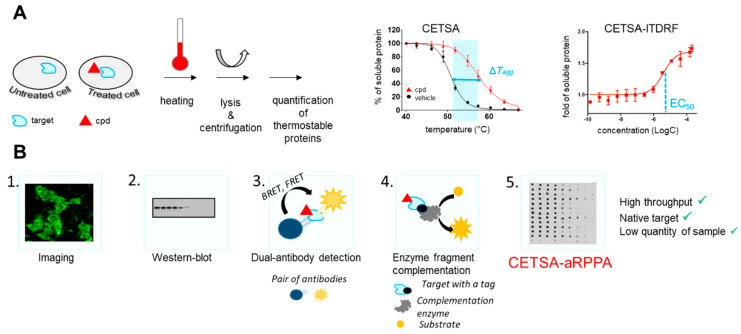
Target engagement using CETSA and CETSA-aRPPA. (**A**) Principle of CETSA and measurement of ΔTagg of protein and ITDRF; (**B**) detection methods: 1. Immunofluorescent staining with a target-directed antibody and high-content imaging; 2. Western blot using immunofluorescent staining; 3. Dual-antibody detection of the folded target protein and antibody proximity detection systems based on, for example, Bioluminescence or fluorescence; 4. enzyme fragment complementation (EFC) system where a small fragment tag (e.g., 42 amino acid of β-galactosidase; or 14 amino acid Hibit fragment of nanoluciferase) is tagged to the target of interest, and compound-mediated target stabilization is subsequently detected by the addition of the enzyme acceptor (EA) fragment (e.g., rest of β-galactosidase or NanoLuc) and luminescence reporter; 5. CETSA-aRPPA uses immunostaining detection (dotblot format) and acoustic reverse phase protein array. CETSA-aRPPA combines a high throughput, low quantity of material, and advantageously no need for target tagging, in comparison to other detection methods.

Several techniques have recently emerged, such as cellular thermal shift assay (CETSA), a target engagement tool used in intact cells. CETSA is based on the ligand-induced thermal stabilization of the targeted protein that causes a shift (also referred to as thermal shift) in the aggregation temperature (Tagg) of the protein (Figure 2A) [11]. It can be used to guide lead optimization [12]. It is now routinely included in screening cascades for both hit and target validation and it offers several advantages [13]. For example, it allows consolidating results from an on-target primary screen with elements such as target engagement by compound and cell-membrane permeability, or characterization of compounds in complex settings where phenotypic screening for the given target is not possible. It also provides a way to validate ligands that may be chaperones, with applications, for example, in rare diseases [13].

Several detection methods are used to quantify the protein bound to the ligand as described in Figure 2B. In order to expand the use of CETSA as a decision tool, efforts have been made on increasing throughputs and detection methods (Figure 2B). Initially, Western blot and imaging were used, but these methods suffered from a low throughput. In order to improve this key parameter, CETSA has been coupled with AlphaLISA^®®^ detection [14] or enzyme fragment complementation (EFC) [15]. Nevertheless, these techniques require specific antibodies to detect the endogenous target protein or the exogenous tagged target protein expression that hinder their general use. As a result, Western blot (WB) is still the most used readout for CETSA, despite the substantial cell amounts and low throughput.

Using a nanoacoustic transfer device, we developed an original CETSA-aRPPA method as a new high-throughput tool to explore target engagement (Figure 2B) [16]. Additionally, apart from measuring the variation of the aggregation temperature (Tagg) in the context of cells in the presence of the compound, it can be used to assess the dose-dependent stabilization of the target-of-interest ITDRF (isothermal dose-response fingerprint), transcribing the potency of the compound to interact with the target.

We use this routinely in our projects to evaluate target engagement. As an example, we studied different insulin degrading enzyme (IDE) inhibitors in hepatocytes and showed the impact of a single fluorine on target engagement in cells [16]. Another application of this protocol is to allow the evaluation of multiple targets at the same time.

## 3. Chemical Biology Strategies to Identify New Hits

### 3.1. Screening of Focused Libraries or Clinical Compounds Libraries on Metalloproteases

Among the different hit-discovery strategies, we classically use high-throughput screening of known drugs and clinical compounds (TEΞLibrary available from the French company Apteeus, located in Lille) or focused libraries [17,18]. We have applied such strategies to the discovery of inhibitors of two atypical metalloproteases, namely insulin degrading enzyme (IDE) and endoplasmic-reticulum aminopeptidase 2 (ERAP2), for which biological roles are not completely defined or which have only a few modulators with poor drug-like properties in the literature.

**Figure 3:**
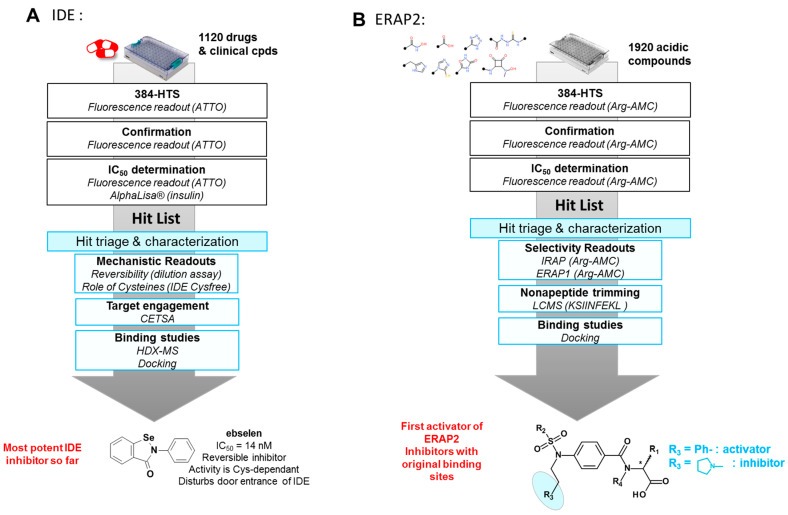
High-throughput screening cascades for the discovery of metalloprotease inhibitors. (**A**) Ebselen is the most active IDE inhibitor. It is a reversible covalent inhibitor that impairs the open–close conformational shift of IDE and the entry of the substrates; (**B**) screening a focused library of acidic compounds allowed the discovery of the first activator of ERAP2 among a family of inhibitors.

IDE is an intriguing metalloprotease from the M16 family [19]. We ran a high-throughput drug repurposing screening of a library of drugs and clinical candidates to identify new drug-like inhibitors with optimal pharmacokinetic properties to probe these roles (Figure 3A). We identified several inhibitors of IDE among which ebselen was the most potent IDE inhibitor described so far (IC_50_ (insulin) = 14 nM). Mechanistic studies suggested ebselen could be a reversible covalent inhibitor of IDE. Biophysical methods such as HDX-MS pointed out how ebselen disturbs the open-closed conformation equilibrium of IDE in a distinct manner to previously described inhibitors [20].

Both the fact that the inhibitory activity of ebselen towards IDE is the highest listed activity on a human target and the proof of IDE engagement by ebselen in hepatocytes explain some of its reported activities in metabolism, such as its insulin-mimetic action or improved hepatic insulin signaling and restored glucose tolerance in vivo.

ERAP2, another metalloprotease from the M1 family (aminopeptidase), trims peptides for their presentation by MHC-I proteins. Polymorphisms of this enzyme have been linked to the risk of developing several pathologies including autoinflammatory diseases, infections, and cancers [21,22]. To date, only a few ERAP2 inhibitors have been identified, but these compounds either lack selectivity against other metalloenzymes and/or show poor drugability properties. We recently screened our 2000-member library of acidic compounds on ERAP2 to find alternative scaffolds [23].

We coupled screening on a small substrate with hit triage using longer antigens (nonapeptides) and selectivity screening against closely related aminopeptidases (ERAP1 and IRAP). Thanks to this screening cascade (Figure 3B), we identified 11 inhibitors of ERAP2. In particular, we discovered a series of carboxylic acids that behave either as inhibitors or as activators of small substrates hydrolysis [23]. Interestingly, these compounds bind the catalytic site but shape it for optimized binding and hydrolysis of small substrates like Arg-AMC (activators) or for preventing longer peptides to bind (inhibitors).

Screening has thus allowed us to identify new modulators of metalloproteases with atypical binding modes or pharmacological activities.

### 3.2. Kinetic Target Guided Synthesis

**Figure 4:**
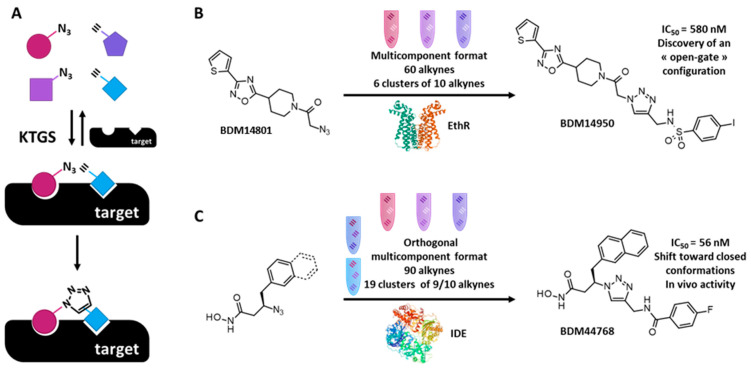
(**A**) Principle of kinetic target-guided synthesis (KTGS) with the example of the in situ click chemistry strategy. A pool of azides and alkynes are presented to the protein of interest, which stabilizes a pair of affine reagents in reacting configuration (close proximity and proper orientation of their compatible reactive moieties). The protein accelerates the irreversible reaction of these two reagents to afford the final 1,2,3-triazole ligand; (**B**) KTGS by EthR using a multicomponent strategy from one azide and 60 diverse alkynes leading to BDM14950 that traps a new “open-gate” conformation; (**C**) KTGS by IDE using an orthogonal multicomponent strategy from two azides and 90 diverse alkynes leading to 66 hits including the best in vivo active IDE inhibitor BDM44768 that shifts IDE conformer ensemble toward closed conformations.

Among the novel bioorthogonal chemical breakthroughs in chemical biology and drug discovery, protein-templated synthesis is an uncommon and, to some extent, an unexplored strategy. Dynamic combinatorial chemistry (DCC) [24] and kinetic target-guided synthesis (KTGS) use the targeted protein as a template to create its own ligands from biocompatible and reactive reagents. In KTGS, the irreversible reaction between the reagents occurs following their binding to the targeted protein that brings them in close proximity and properly orients their compatible reactive moieties (Figure 4A) [25,26,27]. Azides and alkynes are the biocompatible reagents, which are the most employed and produce protein-templated triazoles by in situ click chemistry, a class of KTGS. Triazoles are appealing scaffolds in drug discovery as they can be involved in numerous interactions (e.g., dipolar interactions, H-bond interactions, aromatic interactions) [28,29]. Thus, with its tremendous potential, in situ click chemistry has drawn our attention and subsequently was integrated in our array of strategies for our drug discovery programs.

First, in a project related to the mycobacterial transcriptional regulator EthR, the thienoacetyl group of an ‘in-house’ weak inhibitor was replaced by an acetylazido group affording BDM14801 [30]. Sixty diversified alkynes were selected from our dedicated library for their possible capability to bind in a hydrophobic pocket of EthR close to the ligand binding domain where the azido moiety of BDM14801 interacts according to X-ray analysis. Then, the KTGS was performed in a multicomponent format, where the alkynes were displayed to the protein as a mixture of competing reagents. This multicomponent format has the advantage of reducing the number of reaction wells and thus decreasing the amount of enzyme and the duration of analytical screening compared to the binary format. For the EthR-templated KTGS, the azide was incubated with 6 clusters of 10 alkynes in the presence of the target. Remarkably, after 24 h of incubation, SIM LC/MS analysis identified one hit: the 1,4-disubstituted 1,2,3-triazole BDM14950 (Figure 4B). This compound proved to be a submicromolar inhibitor (IC_50_ = 580 nM). Moreover, thanks to this KTGS experiment, a new “open-gate” conformation of EthR was discovered with the flip of two mobile phenylalanines induced by the formation of the triazole ligand (PDB code: 3O8H). This phenomenon affected the transcriptional repressor activities of EthR and allowed the access of a new hydrophobic region that could be further explored for the discovery of new inhibitors.

Another successful KTGS experiment was performed on insulin degrading enzyme (IDE) [31]. Two azides were rationally designed thanks to information available on substrate preference and known inhibitors. These azides bore an hydroxamate moiety as a warhead to coordinate the zinc ion in the N-terminal domain (IDE-N). Ninety varied alkynes were picked out of our dedicated library for their potential ability to bind the C-terminal domain (IDE-C) of the catalytic site. The KTGS was carried out in an orthogonal multicomponent format where the alkynes were arranged in orthogonal clusters in function of the type of their backbone or their substituents. In this sorting strategy, any alkynes compete with its counterparts in two different competing environments. This format has the advantages of maximizing the chance of templated triazole formation and reducing the rate of false negatives. For our KTGS experiment involving IDE, the two azides were incubated individually with the metalloprotease and 19 clusters of 9 or 10 alkynes. Of note, the hit rate was quite good (18.3%) and 1,4- and 1,5-disubstituted triazoles were detected by LC-MS-TOF analysis. Impressively, among the 66 hits, the KTGS delivered BDM44768 (Figure 4C), the first inhibitor targeting the catalytic site with an in vivo activity that allowed us to invalidate this metalloprotease as a target to treat type-2 diabetes. Moreover, co-crystallization of this compound with IDE revealed that it interacted with both IDE-N and IDE-C terminal domains, shifting the metalloprotease towards its closed, inhibited conformation (PDB code: 4NXO). Consequently, KTGS showed its proficiency to explore unknown or less abundant conformations.

### 3.3. Fragment-Based Drug Discovery

**Figure 5:**
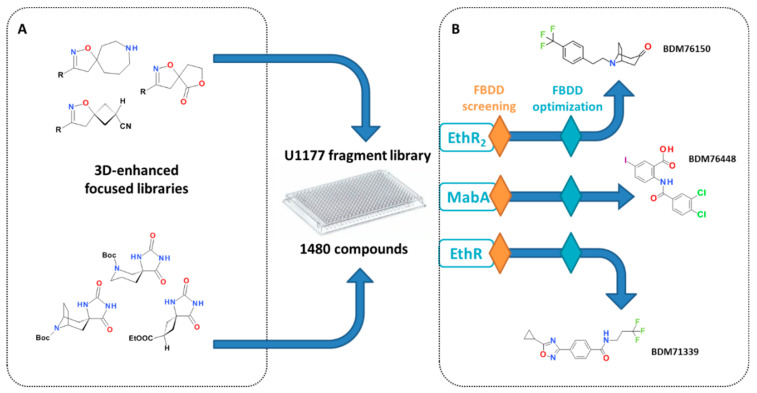
Fragment-based drug design (FBDD) approaches used to identify hits in three different anti-infectious drug discovery programs. (**A**) Enrichment of the library with 3D-fragments; (**B**) structures of three optimized inhibitors of mycobacterial proteins EthR_2_, MabA and EthR.

In the last two decades, fragment-based approaches have gained a lot of attention alongside the discovery of new biologically active molecules, notably for their capacity to explore new chemical spaces against very challenging targets [32]. Fragment-based drug design relies on the screening of small molecules heavy atom count (HAC < 17–20), so-called fragments, compared to lead-like and drug-like molecules (25 < HAC < 35) found in high-throughput screening libraries. Using fragments allows for a better sampling of the chemical space, even with small libraries (usually 10^3^–10^4^ compounds) [33,34] which are less costly to assemble and use and are therefore more accessible to small biotechs and academics. Another advantage with fragments is that they usually display more efficient binding modes [35]. However, because they create fewer interactions with the target, the hits from the screening tend to have low affinities (1 mM–10 µM) and need to be extended to create new interactions and increase their potency. This optimisation of the fragments hit can be performed using three main strategies: fragment-growing, fragment-linking and fragment-merging. Among these, fragment-growing is the most common and simply consists of “growing” the initial hit fragment by adding new chemical moieties in order to create new interactions with the target. This process is highly facilitated when binding mode information is available. Fragment-linking involves the assembly of two fragments that bind in non-overlapping binding sites. Finally, fragment merging is the merger of structural aspects of fragments overlapping with other known ligands. One of the main criteria for the success of a fragment-based approach is the quality of the initial fragment-library. It has to be well designed to largely explore the available chemical space and maximize the chances of hit-identification. One of the main shortcomings in current chemical libraries is the lack of three-dimensionality, which has been proven to lead to improved optimization [36,37]. We designed original, easily accessible, and chemically tractable spirocyclic scaffolds. A 3D-enhanced focused library of 50 spiroisoxazolines [38] and spirohydantoins [39] was therefore synthesized to enrich our in-house fragment library (Figure 5A).

Due to their small size, fragments usually display excellent physicochemical properties, a very attractive feature in the aim of penetrating the thick and poorly permeable *M. tuberculosis* cell envelope, for instance [40]. Therefore, in the scope of our anti-infectious drug discovery programs, we applied fragment-based approaches for the discovery of inhibitors of transcription factors EthR, EthR_2_, and the mycobacterial enzyme MabA, part of the fatty acid elongation system FAS-II (Figure 5B).

#### 3.3.1. Discovery of Fragments Targeting EthR and EthR_2_

By combining surface plasmon resonance (SPR) and X-ray crystallography, we identified 4-iodophenylsulfonamide as a weak binder of the mycobacterial transcriptional repressor EthR [5]. The simultaneous presence of two fragment entities in the binding pocket allowed for parallel fragment-growing, fragment-linking, and fragment-merging strategies. Although both fragment-linking and fragment-merging strategies allowed the exploration of previously undescribed binding pockets, the fragment-growing approach proved to be the most efficient and quickly delivered low nanomolar ethionamide boosters. Multiparametric and structure-guided optimization led to BDM71339 (Figure 5B), the first fragment-based EthR inhibitor with in vivo activity in a mice model of tuberculosis infection [9].

As previously mentioned, an alternative ethionamide bioactivation pathway, regulated by a second transcriptional regulator EthR_2_, has been identified [10]. The success encountered with the fragment-based approach for the identification of EthR inhibitors encouraged us to start from fragments to identify new chemotypes of EthR_2_ ligands. The screening of EthR_2_ with our fragment library using thermal shift assay led to the identification of five new chemical series [41]. Structure-based optimisation of the tropinone-based scaffold led to BDM76150 (Figure 5B), a sub-micromolar EthR_2_ inhibitor.

#### 3.3.2. Discovery of Fragments as Inhibitors of MabA

Inhibitors of EthR and EthR_2_ boost the bacterial bioconversion of ETH into an ETH-NAD adduct which ultimately inhibits InhA, an enzyme of the fatty acid biosynthesis FAS-II system in charge of elongation of mycolic acids. In addition to InhA, three other enzymes are involved in the FAS-II system: MabA (FabG1), HadAB/BC, and KasA/B. Among all these essential enzymes, only MabA had not yet been investigated for specific inhibitors. We therefore decided to implement a fragment-based approach for the discovery of the first inhibitors [42]. LCMS-MS-based biochemical assay allowed for the identification of six chemical series of MabA inhibitors. Out of these, the anthranilic acid-based family was selected for further optimization. Chemical exploration around the anthranilic acid moiety led to the low micromolar inhibitor BDM76448 (Figure 5B). Affinity for MabA of BDM76448 fluorinated analogues was confirmed by ^19^F-NMR. Indeed, signal perturbations correlated well with IC_50_ measured in the biochemical assay. BDM76448 represents the first example of a MabA specific inhibitor. Further optimization of this compound and validation of its antibacterial mechanism of action is currently ongoing.

## 4. Chemical Biology Approaches to ADME Properties Modulation

### 4.1. Controlling Target Engagement by Innovative Molecular Engineering

Drug safety has been an increasing concern in drug discovery. Improving its selectivity is a key approach to improving the therapeutic index of a drug. Two kinds of selectivities have been widely studied: (1) the drug/target selectivity to minimize potential off-target toxicity and (2) the tissue (organ) selectivity to avoid toxicity resulting from on-target toxicity in an undesired tissue.

**Figure 6:**
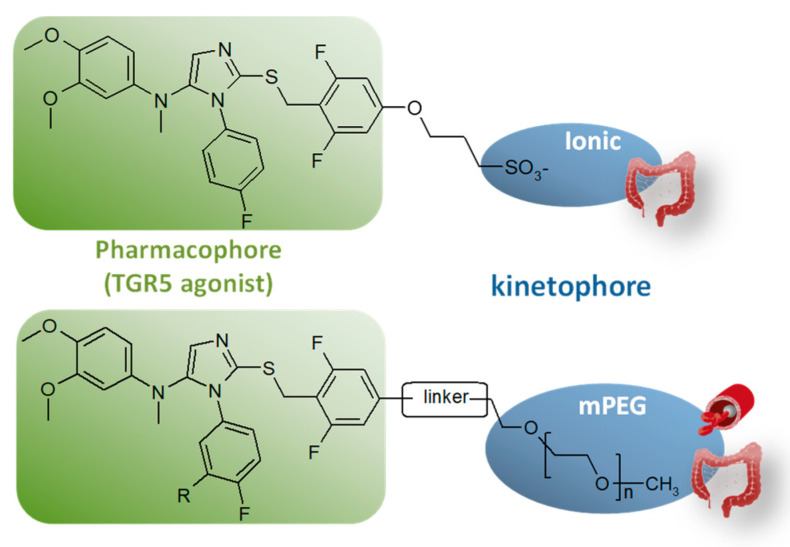
Chimeric TGR5 agonists with fine-tuned pharmacokinetic behavior. For the ionic kinetophores (**top**), compounds with gut-restricted exposure were obtained. In the case of mPEG kinetophores (**bottom**), depending on the length of the PEG moiety tethered on the pharmacophore, either systemic or gut-restricted compounds were obtained.

Targeting the molecule to the desired tissue while minimizing exposure of the rest of the body seemed particularly suitable for the development of agonists of the bile acid receptor TGR5. Indeed, gut-restricted TGR5 agonists would enable the stimulation of GLP-1 secretion by the enteroendocrine L-cells expressing TGR5 without triggering any other unwanted TGR5-related effect. In this context, we targeted our agonists to the intestine through innovative rational molecular engineering. To do so, we used appropriate physicochemical properties outside the oral systemic drug space to obtain high retention within the gut. To access such non-absorbable compounds, our TGR5 agonists were designed as chimeric compounds composed of an optimized TGR5 pharmacophore linked to a highly polar and/or large chemical moiety—the kinetophore—meant to prevent absorption through the intestinal epithelium (Figure 6). Introduced in 2006 [43,44], the kinetophore concept consists in a large and/or highly polar chemical moiety that is linked to a pharmacologically active structure (pharmacophore) to drastically modify its pharmacokinetic properties. Amongst the possible kinetophore moieties, we decided to explore: (1) ionic kinetophores, especially permanently ionized quaternary ammonium and sulfonate groups [45]; and (2) linear methoxy PEG (mPEG) of various lengths (220 to 5 000 Da) [46].

In the first case, modification of a potent TGR5 agonist with a sulfonate moiety (compound 24, Table 1) was proven to be a successful strategy to obtain a robust local target engagement in the not easily accessible distal part of the gut. As might be expected from the very low in vitro permeability of this compound, a low systemic exposure ([C]_plasma_max, Table 1) was measured in plasma and fecal recovery was quantitative (Table 1). Thanks to its fine-tuned pharmacokinetic behavior (Table 1), this TGR5 agonist efficiently stimulates enteroendocrine L-cells located in the lower intestinal tract after oral dosing while creating a potency window between the efficacious dose evaluated in a glucose tolerance test and the dose triggering an unwanted gallbladder response.

As for the mPEG kinetophore, our study demonstrated that increasing the chain length in our PEGylated conjugates expectedly altered their physicochemical properties such as aqueous solubility and lipophilicity and decreased their susceptibility to oxidative metabolism and their passive permeation through cell membranes (Table 2). Finally, the in vivo pharmacokinetic studies have shown that anchoring an mPEG of suitable length to our TGR5 pharmacophore allows one to balance intestinal absorption and hepatic metabolism to obtain either intestine-restricted or systemic TGR5 agonists. Consistent with its permeability (Table 2), compound P7 displays a very low exposure of the intestine after oral administration that could be the result of early and efficient gut absorption. In contrast, high concentrations (>500 μM) of the longer PEG conjugates (P9, P11, and P12) were measured in the intestine, in line with their very low permeability. Interestingly, despite its low permeability and its high molecular weight, P9 displays a higher plasma exposure than compound P7. Even if P7 is expected to be more consistently absorbed with its higher permeability, the better bioavailability of P9 could be the result of its better metabolic stability (as observed with the low intrinsic clearance measured in vitro on microsomes, Table 2).

### 4.2. Rationalizing and Optimizing Plasma Stability

For medicinal chemists, plasma stability is an essential criterion to optimize compounds that are unstable in plasma and tend to display poor oral bioavailability and thus poor or undetectable activity, though they may be very potent in vitro.

The hydroxamic acid function is mainly hydrolysed to the corresponding carboxylic acid, in particular in rodents, whose plasma is more aggressive due to the presence of specific esterases [47]. Unfortunately, this metabolite is usually much less active on the target and has different ADME properties. Thus, the transformation of this key pharmacophoric element compromises the development potential of some hydroxamic acids and the obtainment of proof of concept in rodents.

**Figure 7:**
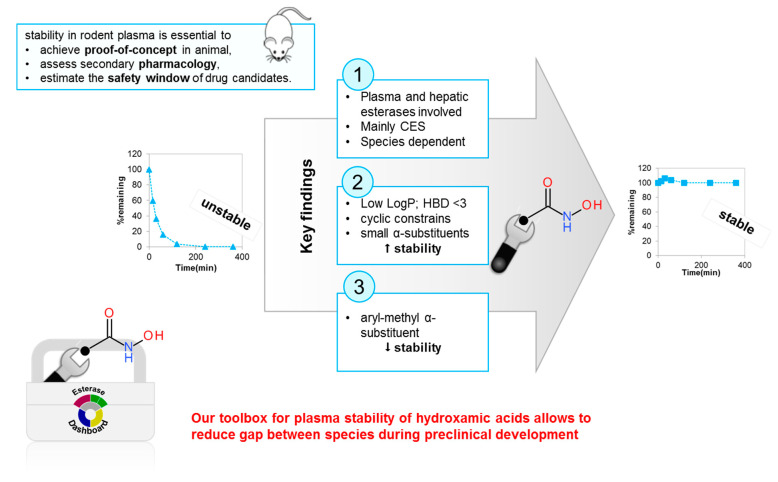
Use of pharmacokinetic studies to provide toolbox for optimization: the case of hydroxamic acids. CES: carboxylesterases, LogP: partition coefficient; HBD: count of hydrogen-bond donors.

We developed assays to measure the plasma stability of hydroxamic acids and identify the esterases involved in hydrolysis (Figure 7) [48]. In addition, we identified all key modifications to enhance the stability by finetuning the arrangement of substituents around the electrophilic carbonyl of the hydroxamate function (Figure 7).

### 4.3. Controlling the Cell Clock In Vivo with an Antedrug to Understand a Clinical Observation

**Figure 8:**
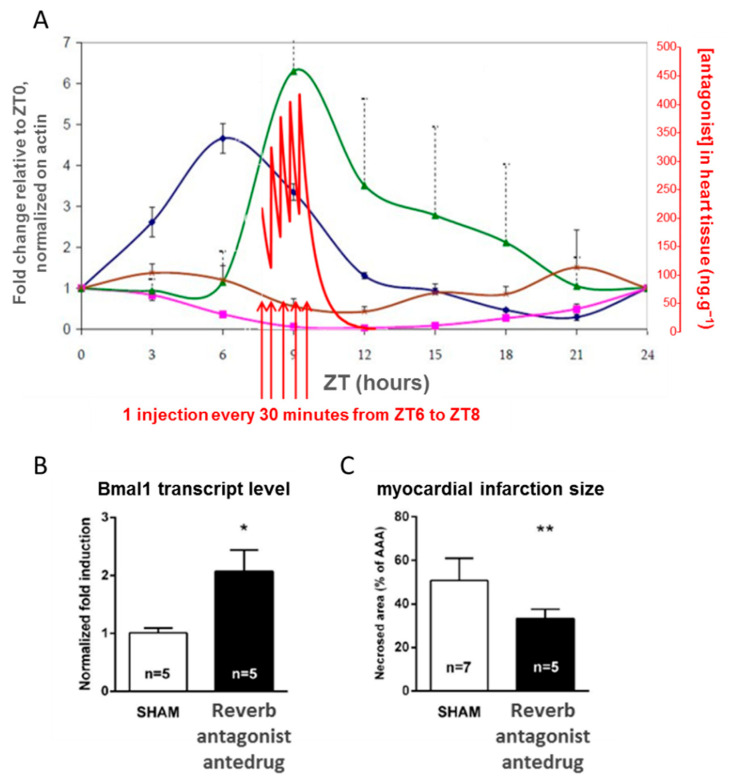
Rat model of perioperative cardiac injury. Panel (**A**): in blue and pink: plots of the fold change of respectively RevErb and Bmal1 gene expressions relative to ZT0, in green and brown: plots of the fold change of respectively RevErb and p21 proteins relative to ZT0. In red: plot of the plasma concentration of the RevErb antagonist. ZT means Zeitgeber Time. ZT0 is set at the beginning of the light phase. Five bolus injections of the antagonist provide an exposure profile matching the target protein expression. Panels (**B**) and (**C**): effects of the pharmacological pulse with the RevErb antagonist antedrug: increase of Bmal1 target gene (Panel (**B**)), and reduction in the infarct size (Panel (**C**)); ** *p* < 0.01 and * *p* < 0.05 vs SHA.

In this project, we illustrate the concept of antedrug to downregulate a pharmacological pathway that is naturally oscillating between two states during the circadian circle. An antedrug is defined as a pharmacologically active compound designed to undergo biotransformation to the readily excretable inactive form upon entry in the systemic circulation. With such a compound, it is theoretically possible to accurately control the time of target engagement. Montaigne et al. showed that the occurrence of perioperative myocardial injury in cardiac surgery is linked to the time of the intervention: morning surgery is more likely to be rapidly followed by a major cardiac event than afternoon surgery [49]. Long-term adverse events are also more likely in the group of patients undergoing morning surgery. A study of gene expression in heart cells from patients showed that the expression in the morning and in the afternoon of Rev-Erb, a nuclear receptor involved in the circadian clock were significantly different. To demonstrate the causal relationship between the state of the circadian clock in the heart and the chance of developing short- or long-term perioperative cardiac injury, we proposed to study the effect of a short-lived Rev-Erb antagonist (an antedrug) in a rat model of cardiac surgery. In this model, an acute treatment of rats before surgery with the antagonist actually resets the heart clock and lowers the extent of perioperative injury. As the pharmacological intervention specifically targeting the clock pathway precedes the observation, the causal relationship can be experimentally established. Of note, the target protein RevErb displays large circadian variations as it negatively regulates the transcription of its own coding gene. To further support our conclusions on the mechanism of the clinical observation, we also checked that the antagonist antedrug timely engages the RevErb pathway by checking, on the one hand, that it reaches pharmacologically active concentrations when the target protein is at its highest level in the heart and, on the other hand, by quantifying the target protein as well as the target genes following treatment. These data are presented in Figure 8. For once, the short half-life of the antagonist was an advantage to create a short pharmacological pulse and reset the clock of the targeted organ.

## 5. Conclusions

In this paper, we show how synthetic, structural, and analytical chemical concepts can be used to (1) identify new pathways and targets of therapeutic interest, (2) specifically target a class of proteins and selectively bind to one of its members, (3) control the compound half-life and diffusion through specific biophases of the organism, (4) and assess ligand target interactions in cells. The few examples chosen here show that small molecules have desirable properties for drug discovery and chemical biology, e.g.,: vast diversity, ability to cross cell membranes, many binding modes, chemical tractability.

Due to the complexity and adaptability of biological systems and the residual ability of pharmacological tools and drugs to bind to secondary targets, it must be remembered that the knowledge held on drug candidates usually remains incomplete, even at the clinical stage. Therefore, thorough observations of drug effects in various models during and after development can always provide new insights into biological systems, making drug discovery and chemical biology approaches not always distinguishable.

## Figures and Tables

**Table 1:** Pharmacological and ADME/PK properties of a sulfokinetophore-coupled TGR5 agonist (cpd 24) compared to the parent agonist (cpd 3) [45].

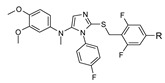	**R**	**H**	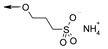
	**Cpd**	**3**	**24**
	In vitro activity		
	hTGR5 EC_50_ (nM)	35	24
	mTGR5 EC_50_ (nM)	0.8	0.4
	In vitro ADME properties		
	Solubility (µM) ^a^	6.3	>200
	LogD (7.4) ^b^	3.5	0.8
	P_app_ A-B ^c^	5.32	0.031
	P_app_ B-A ^c^	5.47	19
	Efflux ratio ^c^	1	636
	Cl_int_ ^d^	1287	1211
	In vivo PK properties ^e^		
	[C]_plasma_max (nM)	NT	102
	Fecal recovery (%) ^f^	NT	100

^a^ solubility < 10µM: low to moderate solubility, solubility > 100 µM: high solubility. ^b^ Lipophilicity is a physicochemical parameter that has a significant influence on various PK properties. Hydrophilic compounds (LogD < 0) usually are highly soluble but exhibit low permeability across the gastrointestinal tract. Highly lipophilic compounds (Log D > 5) may exhibit issues such as low solubility, metabolic instability, high plasma protein binding. LogD between 0 and 3 is usually expected to provide a good balance between solubility and permeability and tends to be optimal for oral absorption. ^c^ Cell membrane permeability assessed on a Caco-2 cell monolayer. “A-B” indicates the transport from apical side to basolateral side. “B-A” indicates the transport from basolateral side to apical side. Permeability is expressed in 10^−6^ cm/s. Permeability classification: low: P_app_ < 2 × 10^−6^ cm/s; high: P_app_ > 20 × 10^−6^ cm/s. Efflux ratio: ratio of P_app_ B-A/P_app_ A-B. When a compound has an efflux ratio greater than 2, it suggests that the compound may be subject to active efflux. ^d^ Cl_int_: intrinsic clearance measured on male mouse microsomes (µL/min/mg proteins). ^e^ Compounds were administered orally (20 mg/kg, formulated in Tween 0.1%). n = 3 mice/time; male C57Bl6/J mice. ^f^ Feces collected 24 h after compound dosing. Compound was extracted with organic solvent and analyzed quantitatively using mass spectrometry. NT: not tested.

**Table 2:** In vitro pharmacological, physico-chemical and ADME parameters of PEGylated compounds (P7, P9, P11, P12) compared to the parent agonist (Cpd2) [46].

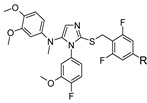	**R**	**H**	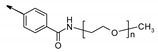			
	**Cpd**	**2**	**P7**	**P9**	**P11**	**P12**
	n (PEG unit)	-	4–13	11–25	31–57	100–138
	MW (g/mol)	516	824–1220	1132–1748	2012–3156	5048–6720
	In vitro activity					
	hTGR5 EC_50_ (nM)	20	60	145	515	1102
	mTGR5 EC_50_ (nM)	0.8	5	13	25	63
	In vitro ADME properties					
	Solubility (µM) ^a^	8.8	151	>200	>200	>2 00
	LogD_7.4_	3.97	3.42	1.58	−1.53	−1.58
	Cl_int_^b^	1254	524	17	8	11
	P_app A-B_^c^	9.3	4.9	<0.02	<0.12	NT
	P_app B-A_^c^	8.5	19.5	9.1	<0.03	NT

^a^ Solubility < 10µM: low to moderate solubility, solubility > 100µM: high solubility. ^b^ Cl_int_: intrinsic clearance measured on male mouse microsomes (µL/min/mg proteins). ^c^ Permeability on a Caco-2 cell monolayer. “A-B” indicates the transport from the apical side to the basolateral side; “B-A” indicates the transport from the basolateral side to the apical side. Permeability is expressed in 10^−6^ cm/s. Permeability classification: low, P_app_ < 2 × 10^−6^ cm/s; high, P_app_ > 20 × 10^−6^ cm/s. Efflux ratio: ratio of P_app_ B-A/P_app_ A-B. When a compound has an efflux ratio greater than 2, it suggests that the compound may be subject to active efflux. NT: not tested.

## Data Availability

Not available.

## References

[1] Laborde J., Deraeve C., Bernardes-Génisson V. (2017). Update of Antitubercular Prodrugs from a Molecular Perspective: Mechanisms of Action, Bioactivation Pathways, and Associated Resistance. ChemMedChem.

[2] Morlock G.P., Metchock B., Sikes D., Crawford J.T., Cooksey R.C. (2003). ethA, inhA, and katG Loci of Ethionamide-Resistant Clinical Mycobacterium tuberculosis Isolates. Antimicrob. Agents Chemother..

[3] Baulard A., Betts J., Engohang-Ndong J., Quan S., McAdam R.A., Brennan P.J., Locht C., Besra G. (2000). Activation of the Pro-drug Ethionamide Is Regulated in Mycobacteria. J. Biol. Chem..

[4] Willand N., Dirié B., Carette X., Bifani P., Singhal A., Desroses M., Leroux F., Willery E., Mathys V., Deprez-Poulain R. (2009). Synthetic EthR inhibitors boost antituberculous activity of ethionamide. Nat. Med..

[5] Villemagne B., Flipo M., Blondiaux N., Crauste C., Malaquin S., Leroux F., Piveteau C., Villeret V., Brodin P., Villoutreix B.O. (2014). Ligand Efficiency Driven Design of New Inhibitors of Mycobacterium tuberculosis Transcriptional Repressor EthR Using Fragment Growing, Merging, and Linking Approaches. J. Med. Chem..

[6] Flipo M., Desroses M., Lecat-Guillet N., Dirié B., Carette X., Leroux F., Piveteau C., Demirkaya F., Lens Z., Rucktooa P. (2011). Ethionamide Boosters: Synthesis, Biological Activity, and Structure−Activity Relationships of a Series of 1,2,4-Oxadiazole EthR Inhibitors. J. Med. Chem..

[7] Flipo M., Desroses M., Lecat-Guillet N., Villemagne B., Blondiaux N., Leroux F., Piveteau C., Mathys V., Flament M.-P., Siepmann J. (2011). Ethionamide Boosters. 2. Combining Bioisosteric Replacement and Structure-Based Drug Design To Solve Pharmacokinetic Issues in a Series of Potent 1,2,4-Oxadiazole EthR Inhibitors. J. Med. Chem..

[8] Bernard C., Willand N., Déprez B., Jarlier V., Baulard A., Veziris N. EthR inhibitor BDM41906 boosts the in vivo antituberculous activity of ethionamide in a murine model. Proceedings of the 22th European Congress of Clinical Microbiology and Infectious Diseases.

[9] Villemagne B., Machelart A., Tran N.C., Flipo M., Moune M., Leroux F., Piveteau C., Wohlkönig A., Wintjens R., Li X. (2020). Fragment-Based Optimized EthR Inhibitors with in Vivo Ethionamide Boosting Activity. ACS Infect. Dis..

[10] Blondiaux N., Moune M., Desroses M., Frita R., Flipo M., Mathys V., Soetaert K., Kiass M., Delorme V., Djaout K. (2017). Reversion of antibiotic resistance inMycobacterium tuberculosisby spiroisoxazoline SMARt-420. Science.

[11] Comess K.M., McLoughlin S.M., Oyer J.A., Richardson P.L., Stöckmann H., Vasudevan A., Warder S.E. (2018). Emerging Approaches for the Identification of Protein Targets of Small Molecules—A Practitioners’ Perspective. J. Med. Chem..

[12] Lundgren S. (2019). Focusing on Relevance: CETSA-Guided Medicinal Chemistry and Lead Generation. ACS Med. Chem. Lett..

[13] Henderson M.J., Holbert M.A., Simeonov A., Kallal L.A. (2019). High-Throughput Cellular Thermal Shift Assays in Research and Drug Discovery. SLAS Discov. Adv. Life Sci. R D.

[14] Shaw J., Leveridge M., Norling C., Karén J., Molina D.M., O’Neill D., Dowling J.E., Davey P., Cowan S., Dabrowski M. (2018). Determining direct binders of the Androgen Receptor using a high-throughput Cellular Thermal Shift Assay. Sci. Rep..

[15] Martinez N.J., Asawa R., Cyr M.G., Zakharov A., Urban D.J., Roth J.S., Wallgren E., Klumpp-Thomas C., Coussens N.P., Rai G. (2018). A widely-applicable high-throughput cellular thermal shift assay (CETSA) using split Nano Luciferase. Sci. Rep..

[16] Herledan A., Andres M., Lejeune-Dodge A., Leroux F., Biela A., Piveteau C., Warenghem S., Couturier C., Deprez B., Deprez-Poulain R. (2019). Drug Target Engagement Using Coupled Cellular Thermal Shift Assay—Acoustic Reverse-Phase Protein Array. SLAS Discov. Adv. Life Sci. R&D.

[17] Maingot L., Elbakali J., Dumont J., Bosc D., Cousaert N., Urban A., Deglane G., Villoutreix B., Nagase H., Sperandio O. (2013). Aggrecanase-2 inhibitors based on the acylthiosemicarbazide zinc-binding group. Eur. J. Med. Chem..

[18] Charton J., Gauriot M., Guo Q., Hennuyer N., Marechal X., Dumont J., Hamdane M., Pottiez V., Landry V., Sperandio O. (2014). Imidazole-derived 2-[N-carbamoylmethyl-alkylamino]acetic acids, substrate-dependent modulators of insulin-degrading enzyme in amyloid-β hydrolysis. Eur. J. Med. Chem..

[19] Tundo G.R., Sbardella D., Ciaccio C., Grasso G., Gioia M., Coletta A., Polticelli F., Di Pierro D., Milardi D., Van Endert P. (2017). Multiple functions of insulin-degrading enzyme: A metabolic crosslight?. Crit. Rev. Biochem. Mol. Biol..

[20] Leroux F., Bosc D., Beghyn T., Hermant P., Warenghem S., Landry V., Pottiez V., Guillaume V., Charton J., Herledan A. (2019). Identification of ebselen as a potent inhibitor of insulin degrading enzyme by a drug repurposing screening. Eur. J. Med. Chem..

[21] Saulle I., Vicentini C., Clerici M., Biasin M. (2020). An Overview on ERAP Roles in Infectious Diseases. Cells.

[22] Babaie F., Hosseinzadeh R., Ebrazeh M., Seyfizadeh N., Aslani S., Salimi S., Hemmatzadeh M., Azizi G., Jadidi-Niaragh F., Mohammadi H. (2020). The roles of ERAP1 and ERAP2 in autoimmunity and cancer immunity: New insights and perspective. Mol. Immunol..

[23] Medve L., Gealageas R., Lam B.V., Guillaume V., Castillo-Aguilera O., Camberlein V., Piveteau C., Rosell M., Fleau C., Warenghem S. (2021). Modulators of hERAP2 discovered by high-throughput screening. Eur. J. Med. Chem..

[24] Hartman A., Gierse R.M., Hirsch A.K.H. (2019). Protein-Templated Dynamic Combinatorial Chemistry: Brief Overview and Experimental Protocol. Eur. J. Org. Chem..

[25] Bosc D., Camberlein V., Gealageas R., Castillo-Aguilera O., Deprez B., Deprez-Poulain R. (2019). Kinetic Target-Guided Synthesis: Reaching the Age of Maturity. J. Med. Chem..

[26] Bosc D., Jakhlal J., Deprez B., Deprez-Poulain R. (2016). Kinetic target-guided synthesis in drug discovery and chemical biology: A comprehensive facts and figures survey. Futur. Med. Chem..

[27] Oueis E., Sabot C., Renard P.-Y. (2015). New insights into the kinetic target-guided synthesis of protein ligands. Chem. Commun..

[28] Rani A., Singh G., Singh A., Maqbool U., Kaur G., Singh J. (2020). CuAAC-ensembled 1,2,3-triazole-linked isosteres as pharmacophores in drug discovery: Review. RSC Adv..

[29] Agalave S., Maujan S.R., Pore V.S. (2011). Click Chemistry: 1,2,3-Triazoles as Pharmacophores. Chem. Asian J..

[30] Willand N., Desroses M., Toto P.P., Dirié B., Lens Z., Villeret V., Rucktooa P., Locht C., Baulard A., Deprez B. (2010). Exploring Drug Target Flexibility Using in Situ Click Chemistry: Application to a Mycobacterial Transcriptional Regulator. ACS Chem. Biol..

[31] Deprez-Poulain R., Hennuyer N., Bosc D., Liang W.G., Enée E., Marechal X., Charton J., Totobenazara J., Berte G., Jahklal J. (2015). Catalytic site inhibition of insulin-degrading enzyme by a small molecule induces glucose intolerance in mice. Nat. Commun..

[32] Li Q. (2020). Application of Fragment-Based Drug Discovery to Versatile Targets. Front. Mol. Biosci..

[33] Hall R.J., Mortenson P., Murray C.W. (2014). Efficient exploration of chemical space by fragment-based screening. Prog. Biophys. Mol. Biol..

[34] Hann M.M., Leach A.R., Harper G. (2001). Molecular Complexity and Its Impact on the Probability of Finding Leads for Drug Discovery. J. Chem. Inf. Comput. Sci..

[35] Hopkins A.L., Groom C.R., Alex A. (2004). Ligand efficiency: A useful metric for lead selection. Drug Discov. Today.

[36] Lovering F., Bikker J., Humblet C. (2009). Escape from Flatland: Increasing Saturation as an Approach to Improving Clinical Success. J. Med. Chem..

[37] Ritchie T., Macdonald S.J. (2009). The impact of aromatic ring count on compound developability—Are too many aromatic rings a liability in drug design?. Drug Discov. Today.

[38] Tran N.C., Dhondt H., Flipo M., Deprez B., Willand N. (2015). Synthesis of functionalized 2-isoxazolines as three-dimensional fragments for fragment-based drug discovery. Tetrahedron Lett..

[39] Prevet H., Flipo M., Roussel P., Deprez B., Willand N. (2016). Microwave-assisted synthesis of functionalized spirohydantoins as 3-D privileged fragments for scouting the chemical space. Tetrahedron Lett..

[40] Moreira W., Lim J.J., Yeo S.Y., Ramanujulu P.M., Dymock B.W., Dick T. (2016). Fragment-Based Whole Cell Screen Delivers Hits against M. tuberculosis and Non-tuberculous Mycobacteria. Front. Microbiol..

[41] Prevet H., Moune M., Tanina A., Kemmer C., Herledan A., Frita R., Wohlkönig A., Bourotte M., Villemagne B., Leroux F. (2019). A fragment-based approach towards the discovery of N-substituted tropinones as inhibitors of Mycobacterium tuberculosis transcriptional regulator EthR2. Eur. J. Med. Chem..

[42] Faïon L., Djaout K., Frita R., Pintiala C., Cantrelle F.-X., Moune M., Vandeputte A., Bourbiaux K., Piveteau C., Herledan A. (2020). Discovery of the first Mycobacterium tuberculosis MabA (FabG1) inhibitors through a fragment-based screening. Eur. J. Med. Chem..

[43] Kramer W. (2006). Bile Acid Reabsorption Inhibitors (BARI): Novel Hypolipidemic Drugs. Curr. Med. Chem..

[44] Charmot D. (2012). Non-Systemic Drugs: A Critical Review. Curr. Pharm. Des..

[45] Lasalle M., Hoguet V., Hennuyer N., Leroux F., Piveteau C., Belloy L., Lestavel S., Vallez E., Dorchies E., Duplan I. (2017). Topical Intestinal Aminoimidazole Agonists of G-Protein-Coupled Bile Acid Receptor 1 Promote Glucagon Like Peptide-1 Secretion and Improve Glucose Tolerance. J. Med. Chem..

[46] Hoguet V., Lasalle M., Maingot M., Dequirez G., Boulahjar R., Leroux F., Piveteau C., Herledan A., Biela A., Dumont J. (2021). Beyond the Rule of 5: Impact of PEGylation with Various Polymer Sizes on Pharmacokinetic Properties, Structure–Properties Relationships of mPEGylated Small Agonists of TGR5 Receptor. J. Med. Chem..

[47] Du L., Musson D.G., Wang A.Q. (2006). Stability studies of vorinostat and its two metabolites in human plasma, serum and urine. J. Pharm. Biomed. Anal..

[48] Hermant P., Bosc D., Piveteau C., Gealageas R., Lam B., Ronco C., Roignant M., Tolojanahary H., Jean L., Renard P.-Y. (2017). Controlling Plasma Stability of Hydroxamic Acids: A MedChem Toolbox. J. Med. Chem..

[49] Montaigne D., Marechal X., Modine T., Coisne A., Mouton S., Fayad G., Ninni S., Klein C., Ortmans S., Seunes C. (2018). Daytime variation of perioperative myocardial injury in cardiac surgery and its prevention by Rev-Erbα antagonism: A single-centre propensity-matched cohort study and a randomised study. Lancet.

